# Pro-inflammatory and autophagic pathways in lung cancer: dysregulation of key genes ADINR, C/EBPα, CHAST, ATG5, IL-1B, and DICER1-AS1

**DOI:** 10.1016/j.bbrep.2026.102774

**Published:** 2026-09-08

**Authors:** Arezou Sayad, Mahsa Mousazadeh, Mohammad Amin Hahem Nejad, Seyed Soha Momeni, Solat Eslami, Soudeh Ghafouri-Fard

**Affiliations:** a Department of Medical Genetics, School of Medicine, Shahid Beheshti University of Medical Sciences, Tehran, Iran; b School of Medicine, Guilan University of Medical Sciences, Rasht, Iran; c Rajaei Clinical Research Development Unit, Alborz University of Medical Sciences, Karaj, Iran; d Phytochemistry Research Center, Shahid Beheshti University of Medical Sciences, Tehran, Iran; e Department of Medical Biotechnology, School of Medicine, Alborz University of Medical Sciences, Karaj, Iran; f Non-Communicable Diseases Research Center, Alborz University of Medical Sciences, Karaj, Iran

**Keywords:** Lung cancer, lncRNA, *ADINR*, *C/EBPα*, *CHAST*, *ATG5*, IL-1B, *DICER1-AS1*

## Abstract

**Purpose:**

Lung cancer is the second most prevalent malignancy in both sexes and remains the leading cause of cancer-related mortality. This study aimed to evaluate the expression profile of selected Nuclear factor kappa-light-chain-enhancer of activated B cells (NF-κB) signaling-related genes and long non-coding RNAs (lncRNAs), particularly those involved in pro-inflammatory and autophagic pathways, in lung cancer tissues.

**Methods:**

The expression levels of three NF-κB-associated-mRNA coding genes (CCAAT/enhancer-binding protein alpha (C/EBPα), Autophagy Protein 5 (ATG5) and Interleukin-1b (IL-1B)) and six lncRNAs (Antisense Noncoding RNA in the INK4 Locus (ANRIL), Adipogenic Differentiation-Induced Noncoding RNA (ADINR), Cardiac Hypertrophy-Associated Transcript (CHAST), DICER1 Antisense RNA 1 (DICER1-AS1), Hepatocellular Carcinoma Up-regulated Long Non-coding RNA (HULC) and NF-κB Interacting LncRNA (NKILA)) from this pathway were assessed in 42 pairs of lung cancer samples and adjacent non-tumoral tissues. Receiver operating characteristic (ROC) curve analysis was performed to evaluate the diagnostic performance of these transcripts in distinguishing tumoral from non-tumoral samples.

**Results:**

ADINR, C/EBPα, CHAST, ATG5, IL-1B, and DICER1-AS1 were significantly up-regulated in tumor samples compared with adjacent non-tumoral tissues. Among the studied genes, C/EBPα showed the highest level of up-regulation in tumor tissues, with an expression ratio of 412 (95% confidence interval (CI): 159–1075, P < 0.0001). ROC curve analysis showed that the diagnostic accuracy of the evaluated transcripts ranged from 94.12% for C/EBPα to 72.26% for ADINR. The highest positive predictive value and negative predictive value were observed for C/EBPα, at 93.02% and 95.12%, respectively. The Area Under Curve (AUC) values were 0.95 for C/EBPα, 0.84 for IL-1B, 0.79 for CHAST, 0.77 for DICER1-AS1, and 0.74 for ADINR.

**Conclusion:**

The present study indicates up-regulation of NF-κB-related pro-inflammatory and autophagy-associated transcripts in lung cancer tissues. Our findings suggest that these transcripts, particularly C/EBPα, show differential expression patterns that warrant further investigation as candidate tissue biomarkers. However, the translational impact of this study is limited by lack of functional studies. Independent validation and comparison with existing clinical diagnostics are required before any clinical application can be considered.

## Highlights

• C/EBPα showed the strongest up-regulation in lung tumor tissues.
• ADINR, CHAST, ATG5, IL-1B, and DICER1-AS1 were up-regulated.
• C/EBPα distinguished tumor from adjacent tissue with an AUC of 0.95.
• NF-κB-related transcripts may serve as lung cancer tissue biomarkers.

## 1 Introduction

Lung cancer is among the most common cancers and the leading source of cancer death. In fact, it is responsible for the greatest number of cancer-related deaths both in males and females [1]. The majority of lung cancers are non-small cell lung cancer (NSCLC) [2]. Dysregulation of several signaling cascades has been noted in this type of cancer in association with aberrant cell proliferation and drug resistance [3]. Therefore, identification of involved pathogenic pathways in lung cancer has significance in finding novel efficient therapies [[4], [5], [6]]. Among the most important signaling pathways in lung carcinogenesis is the cascade related with the chief cell survival signal, nuclear factor-κB (NF-κB) [7]. This transcription factor (TF) is involved in several steps in lung carcinogenesis and resistance of tumor cells to chemo/radiotherapy [8]. Meanwhile, a handful of long non-coding RNAs (lncRNAs), such as Adipogenic Differentiation-Induced Noncoding RNA (ADINR), Cardiac Hypertrophy-Associated Transcript (CHAST), DICER1 Antisense RNA 1 (DICER1-AS1), and NF-κB Interacting LncRNA (NKILA) have been suggested to be associated with NF-κB, with most of them being dysregulated in malignant tissues [9,10].

We selected six transcripts based on their known or predicted involvement in NF-κB-mediated pro-inflammatory and autophagic pathways, with each chosen for a specific biological rationale: CCAAT/enhancer-binding protein alpha (C/EBPα) — This transcription factor physically and functionally interacts with NF-κB to synergistically induce inflammatory genes [11]. It is also a master regulator of differentiation and has been implicated in various cancers, but its expression pattern in lung cancer tissues relative to adjacent normal tissue remains incompletely characterized. ADINR (CEBPA-DT) lncRNA is transcribed from the upstream region of the C/EBPα gene. Given this regulatory relationship, we hypothesized that ADINR and C/EBPα would show coordinated expression in lung cancer. Interleukin 1B (IL-1B) is a prototypic pro-inflammatory cytokine and a classical downstream target of NF-κB activation. Notably, IL-1B serves as a functional readout of NF-κB pathway activity in the tumor microenvironment [12]. Its expression provides indirect evidence of pathway engagement. Autophagy Protein 5 (ATG5) is a core autophagy gene that has been shown to both regulate and be regulated by NF-κB signaling. Notably, ATG5 can recruit FBXW7 to enhance ubiquitination and degradation of F-box/WD repeat-containing protein 1A, thereby suppressing NF-κB signals [13]. Conversely, ATG5-mediated autophagy has been found to suppress NF-κB signaling to constrain inflammatory responses [14]. However, ATG5 also possesses autophagy-independent functions, including exosome production [15] which might be important in tumor microenvironment. We included ATG5 to explore whether its expression correlates with NF-κB-related transcripts in lung cancer tissues. CHAST has been implicated in NF-κB-associated inflammatory responses and is known to regulate autophagy-related genes. Its expression pattern in lung cancer has not been systematically evaluated. DICER1-AS1 lncRNA has recently been shown to promote colorectal carcinogenesis through the Mitogen-Activated Protein Kinase (MAPK)/Extracellular Signal-Regulated Kinase (ERK) axis [16], a pathway that cross-talks with NF-κB signaling [17]. Its potential role in lung cancer remains unexplored. In addition, we examined three other NF-κB-related lncRNAs (ANRIL, Hepatocellular Carcinoma Up-regulated Long Non-coding RNA (HULC), and NKILA) as part of a broader exploratory analysis. Based on the above rationale, we hypothesize that expression of NF-κB-associated transcripts, including the C/EBPα/ADINR regulatory axis, the pro-inflammatory effector IL-1B, the autophagy-related gene ATG5, and the lncRNAs CHAST and DICER1-AS1, is dysregulated in lung cancer. Thus, we designed the current study to examine the expression of these transcripts in paired lung cancer samples (tumor tissues (TTs) versus normal tissues adjacent to the tumor (NTATs) using real-time Polymerase Chain Reaction (PCR), and to evaluate their potential as candidate tissue biomarkers.

## 2 Materials and methods

### 2.1 Tissues

TT and NTAT samples were acquired from 42 patients diagnosed with NSCLC or carcinoid tumors of the lung. Patients were admitted to Masih-Daneshvari, Khatam or Atieh Hospitals during 2016. Samples were gathered before patients started any kind of radiotherapy or chemotherapy. Tissues were rapidly frozen in liquid nitrogen. The study protocol was approved by the Shahid Beheshti University of Medical Sciences (IR.SBMU.MSP.REC.1395.525). Informed consent forms were signed by all participants/or their guardians.

### 2.2 Expression assays

Total RNA was extracted using the RNJia extraction kit (Roje Technologies, Iran). Production of complementary DNA (cDNA) was executed using the AddScript cDNA Synthesis Kit (ADDBIO, South Korea). Reactions were prepared using RealQ Plus 2x Master Mix Green with high ROX (AMPLIQON, Denmark). Primers sequences were the same as our previous investigations [10,18]. The specificity of these primers was confirmed previously [19]. In addition to the B2M, we used another housekeeping gene (TBP) in our analyses. The following primers were used for amplification of this transcript: Forward primer: GATCTTTGCAGTGACCCAGCATCA; Reverse primer: CTCCAGCACACTCTTCTCAGC.

### 2.3 Statistical analysis

Statistical analyses were conducted using the SPSS version 18.0 (Chicago, IL, USA). Graphs were generated using GraphPad Prism version 9.0 for Windows (La Jolla, CA, USA). Expression level of each gene was compared to NTATs. Expression levels in each sample were calculated using the efficiency-adjusted comparative Ct method, with B2M and TBP as normalizer genes (efficiency-adjusted ΔCt method). Normality of the data distribution was assessed using the Shapiro-Wilk test. Differences in gene expression between tumor tissues and NTATs were evaluated using paired t-tests or the Wilcoxon signed-rank test. Expression ratios with 95% confidence intervals (CIs) were presented. P < 0.05 was considered as significant.

The relative expression levels of genes in lung tumor tissues were quantified using NTATs as the reference. Fold changes were calculated based on this reference and the results were presented as mean of fold change ± 95% confidence interval. To account for multiple comparisons in the nine-gene differential expression analysis, p-values were adjusted using the Benjamini-Hochberg false discovery rate method, and q-values were reported.

Receiver Operating Characteristic (ROC) curves were created to find the optimum -delta Ct cut-off value for each gene. The Youden index was employed to identify cut-off points that best differentiated lung tumor tissues from NTATs. Sensitivity, specificity, accuracy, positive predictive value (PPV), negative predictive value (NPV), and area under the curve (AUC) were calculated based on these cut-offs. ROC analyses were accompanied by 95% CIs for AUC, sensitivity, and specificity. Because the optimal cut-off values were derived and evaluated in the same retrospective cohort, these thresholds should be interpreted as exploratory and require independent validation.

Correlation between gene expression levels was calculated using the Spearman correlation coefficient with the aid of GenEx software (GenEx SW, Multid Analysis AB, Gothenburg, Sweden). Comparisons of gene expression across some clinicopathological variables were made using the Mann-Whitney *U* test. Chi-square test was used to recognize the association between clinical factors and gene expression.

## 3 Results

Table 1 summarizes the features of genes studies in the current study.

**Table 1:** Characteristics of genes studied in this project.

Name/Gene ID	Accession number	Location	Official Full Name	Biotype
CDKN2B-AS1 (ANRIL)	NR_003529.4, NR_047532.2, NR_047533.2, NR_047534.2, NR_047535.2	9p21.3	CDKN2B antisense RNA 1	lncRNA
CEBPA-DT (ADINR)	NR_026887.2	19q13.11	CEBPA divergent transcript	lncRNA
CEBPA (C/EBPα)	NM_001285829.2, NM_001287424.2, NM_001287435.2, NM_004364.5	19q13.11	CCAAT enhancer binding protein alpha	protein coding
ATG5	NM_001286106.2, NM_001286107.2, NM_001286108.2, NM_001286111.2, NM_004849.4, NR_104402.1, NR_104403.1	6q21	autophagy related 5	protein coding
DICER1-AS1	NR_015415.1	14q32.13	DICER1 antisense RNA 1	lncRNA
HULC	NR_004855.3	6p24.3	hepatocellular carcinoma up-regulated long non-coding RNA	lncRNA
IL-1B	NM_000576.3	2q14.1	interleukin 1 beta	protein coding
NKILA	NR_131157.1	20q13.31	NF-kappaB interacting lncRNA	lncRNA

Table 2 summarizes the clinical and demographic characteristics of included cases.

**Table 2:** Clinical characteristics of study participants (N = 42).

Type of cancer	Age (mean ± SD)	Stage	Subtype
NSCLC (N = 37)	58.58 ± 5.93	IA (N = 1)	Adenocarcinoma (N = 18) Squamous Cell Carcinoma (N = 14) Adenosquamous (N = 1) Carcinoid (N = 1) ND (N = 4)
		IB (N = 6)	
		IIA (N = 3)	
		IIB (N = 9)	
		IIIA (N = 7)	
		IIIB (N = 4)	
		IVB (N = 1)	
		ND (N = 6)	
Carcinoid tumors (N = 5)	36.2 ± 11.62	IA (N = 1)	Carcinoid (N = 4)
		ND (N = 4)	ND (N = 1)

ND: Not Determined.

**Fig. 1:**
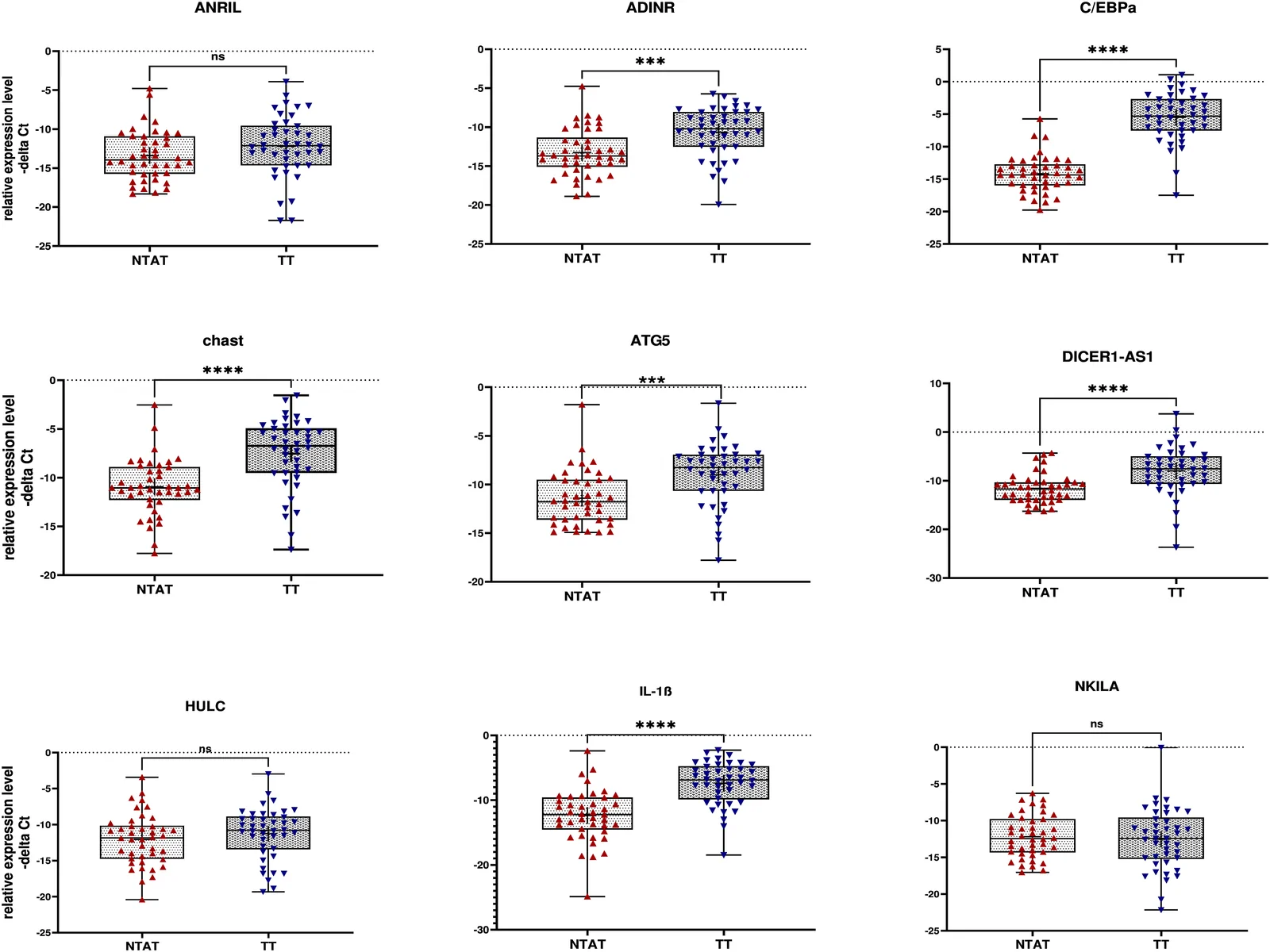
The relative expression levels of nine studied genes in lung tumor tissues (TTs) and normal tissues adjacent to the tumors (NTATs) as described by –delta Ct values (Ct Housekeeping genes- Ct Target gene). Median [line], mean [cross], interquartile range [box], and minimum and maximum values are shown. The data were analyzed using paired *t*-test or Wilcoxon signed-rank test. P < 0.05 was considered significant. Asterisks show significant difference between two groups (*P value < 0.05, **P value < 0.01, ***P value < 0.001, ****P value < 0.0001).

The primary analyses were performed on the combined cohort of 42 paired TT and NTAT samples. Because only five carcinoid cases were available, no separate inferential comparison between carcinoid and NSCLC was conducted. Carcinoid cases were therefore included in the combined analysis, while histological subtype information was reported descriptively. An exploratory comparison was performed only between adenocarcinoma and squamous cell carcinoma within NSCLC. We found significant up-regulation of ADINR, C/EBPα, CHAST, ATG5, IL-1B, and DICER1-AS1 in TT samples compared with NTATs (Fig. 1).

Among the studies genes, C/EBPα was the most up-regulated gene in TTs compared with NTATs (Expression ratio (95% CI) = 412 (159-1075), q-value<0.0001). IL-1B ranked second in this regard with values of 31.3 (10.28-95.5) and <0.0001, respectively. Similarly, DICER1-AS1, CHAST, ADINR and ATG5 were up-regulated in TTs with expression ratios (95% CIs) of 12.2 (4.1-36), 10.57 (3.78-29.54), 6.22 (2.52-15.4) and 5.32 (2.18-13), respectively (q-values of <0.0001, <0.0001, 0.00012 and 0.0004, respectively). Table 3 shows detailed statistics.

**Table 3:** Relative expression of nine studied genes in tumor tissues (TTs) and normal tissues adjacent to the tumors (NTATs). The expression ratio of each gene is shown as mean and 95% Confidence interval.

Gene	Expression ratio	95% CI	P-value	q-value
ANRIL	2.26	0.77-6.6	0.13	0.06
ADINR	6.22	2.52-15.4	**0.0002**	**0.0001**
C/EBPα	412	159-1075	**<0.0001**	**<0.0001**
CHAST	10.57	3.78-29.54	**<0.0001**	**<0.0001**
ATG5	5.32	2.18-13	**0.0007**	**0.0004**
DICER1-AS1	12.2	4.1-36	**<0.0001**	**<0.0001**
HULC	1.74	0.59-5.1	0.29	0.1
IL-1B	31.3	10.28-95.5	**<0.0001**	**<0.0001**
NKILA	0.77	0.26-2.3	0.64	0.21

q-values represent Benjamini-Hochberg adjusted p-values.

Surprisingly, expression levels of all studied mRNA coding genes and lncRNAs were significantly correlated with each other in TTs and NTATs (Table 4). The highest R value (0.83) belonged to ADINR/C/EBPα pair in NTATs, and IL-1B/CHAST and IL-1B/ATG5 pairs in TTs.

**Table 4:** Spearman's correlations between expression levels of studied genes among tumor tissues (TTs) and normal tissues adjacent to the tumors (NTATs). (R values are shown. * Indicates P < 0.05. **indicates P < 0.001).

	ADINR		C/EBPα		CHAST		ATG5		DICER1-AS1		HULC		IL-1B		NKILA	
	NTAT	TT	NTAT	TT	NTAT	TT	NTAT	TT	NTAT	TT	NTAT	TT	NTAT	TT	NTAT	TT
ANRIL	0.75**	0.61**	0.66**	0.67**	0.66**	0.55**	0.64**	0.58**	0.66**	0.59**	0.64**	0.45*	0.78**	0.63**	0.75**	0.61**
ADINR			0.83**	0.66**	0.72**	0.69**	0.61**	0.68**	0.71**	0.42**	0.6**	0.7**	0.71**	0.65**	0.79**	0.79**
C/EBPα					0.7**	0.74**	0.66**	0.73**	0.7**	0.69**	0.58**	0.66**	0.68**	0.74**	0.76**	0.73**
CHAST							0.8**	0.83**	0.74**	0.49**	0.51**	0.56**	0.64**	0.83**	0.72**	0.73**
ATG5									0.69**	0.52**	0.48**	0.66**	0.68**	0.83**	0.72**	0,72**
DICER1-AS1											0.71**	0.57**	0.76**	0.54**	0,78**	0.52**
HULC													0.62**	0.6**	0.61**	0.64**
IL-1B															0.71**	0.8**

**Fig. 2:**
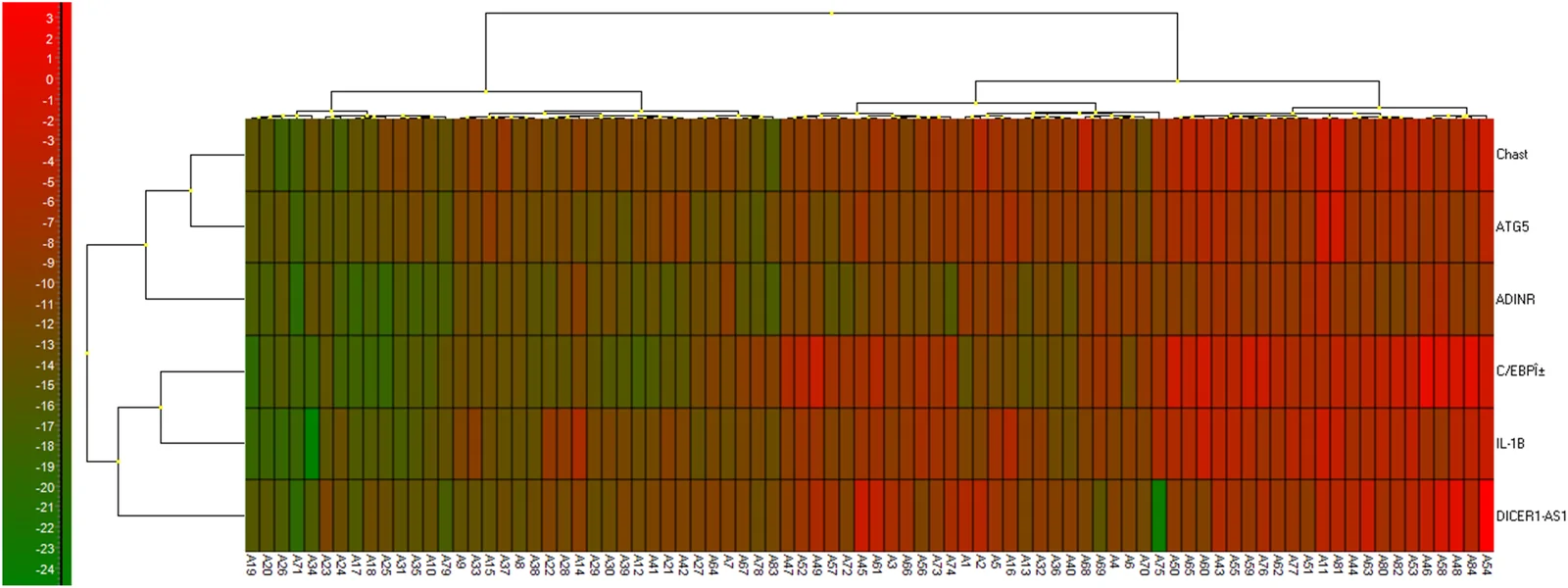
**Heatmap Analysis of Gene Expression in tumor vs. normal tissues.** The heatmap illustrates the expression levels of 6 target genes in 42 paired lung tissue samples, comparing tumor tissues to adjacent normal tissues. Gene expression was analyzed using the -delta Ct (−ΔCt) method, with higher gene expression represented by red and lower expression by green. All six (out of the 9) genes demonstrated significant upregulation in tumor tissues compared to their normal counterparts, suggesting a potential role in tumorigenesis. Data visualization was performed using GenEX software. Genes on the right are clustered using a hierarchical clustering method (Ward's method, Euclidean distances) and 4 clusters were found. Cluster 1 = CHAST and ATG5; Cluster 2 = ADINR; Cluster 3 = C/EBPa and IL-1B; Cluster 4 = DICER-AS1. Most of tumor samples (TT43-TT84) were located on the right side with increased expression of studied genes in this work.

Then, we illustrated expression levels of 6 target genes in 42 paired lung tissue samples in a heatmap. ADINR, C/EBPa, CHAST, ATG5, IL-1B, and DICER1-AS1 genes demonstrated significant upregulation in TTs compared to their normal counterparts, suggesting a potential role in tumorigenesis. Four clusters were found. In brief, CHAST and ATG5 clustered in one group (Cluster 1); Cluster 2 described ADINR levels; Cluster 3 included C/EBPa and IL-1B; and Cluster 4 described DICER-AS1 levels. Most of tumor samples (TT43-TT84) were located on the right side with increased expression of studied genes in this work (Fig. 2).

**Fig. 3:**
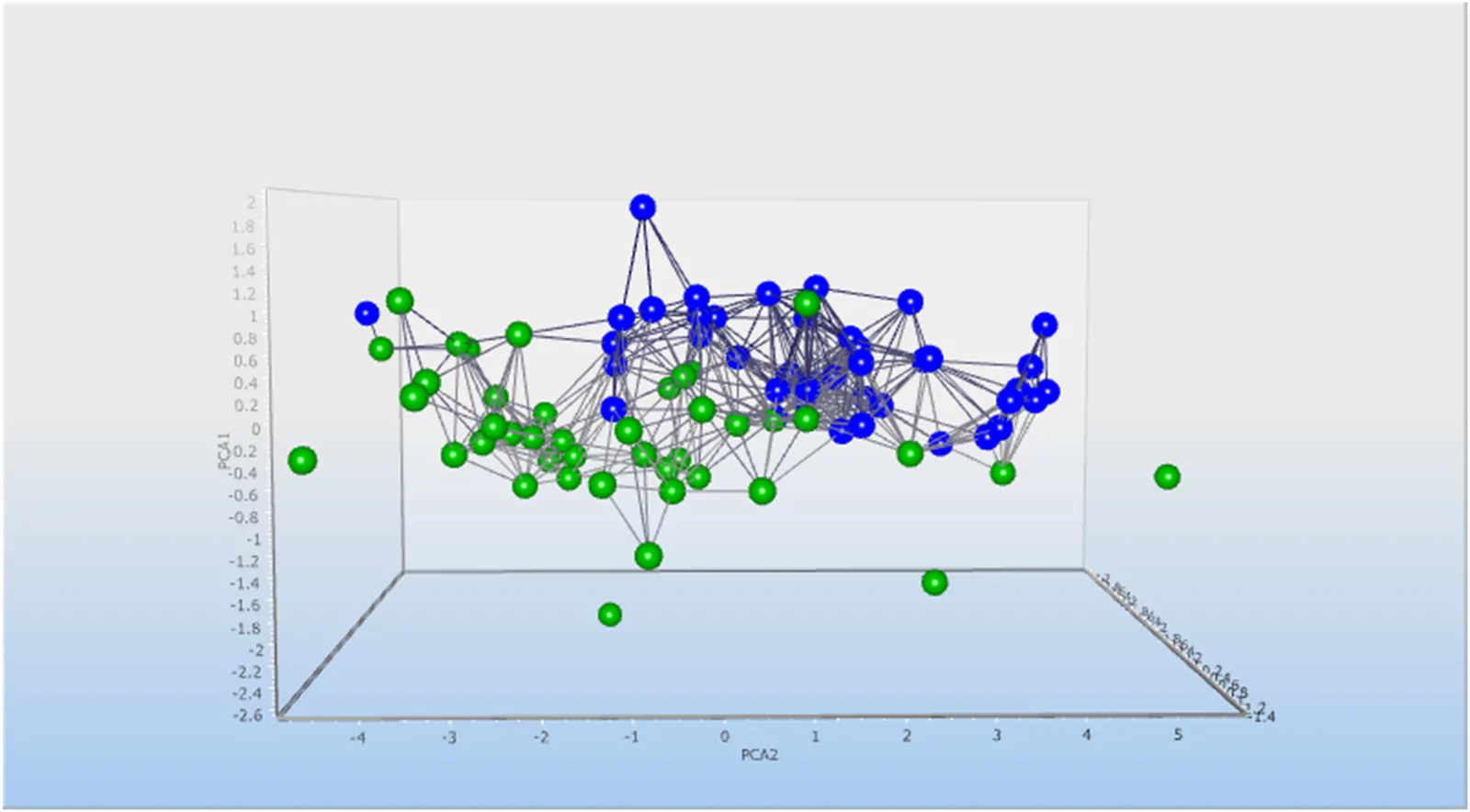
Dynamic principal component analysis (DPCA) of nine studied genes expression profiles. DPCA was used to filter out and exclude genes with low standard deviation. NKILA and HULC were excluded and expression data of other genes were used to cluster samples collected from Normal Tissues Adjacent to the Tumor (blue squares) and lung tumor tissues (green squares) into their corresponding groups. Normalized values were used for PCA.

Then, dynamic principal component analysis (DPCA) was conducted on expression data of studied genes to find how the differentially expressed transcripts were distributed among TT and NTAT samples. DPCA excluded lncRNAs NKILA and HULC with low standard deviation and data of other genes expressions were used to cluster samples collected into their respective groups. In the DPCA plot, NTAT and TT samples showed a visible tendency toward grouping, yet the considerable overlap indicates that the groups are not evidently separated (Fig. 3).

**Fig. 4:**
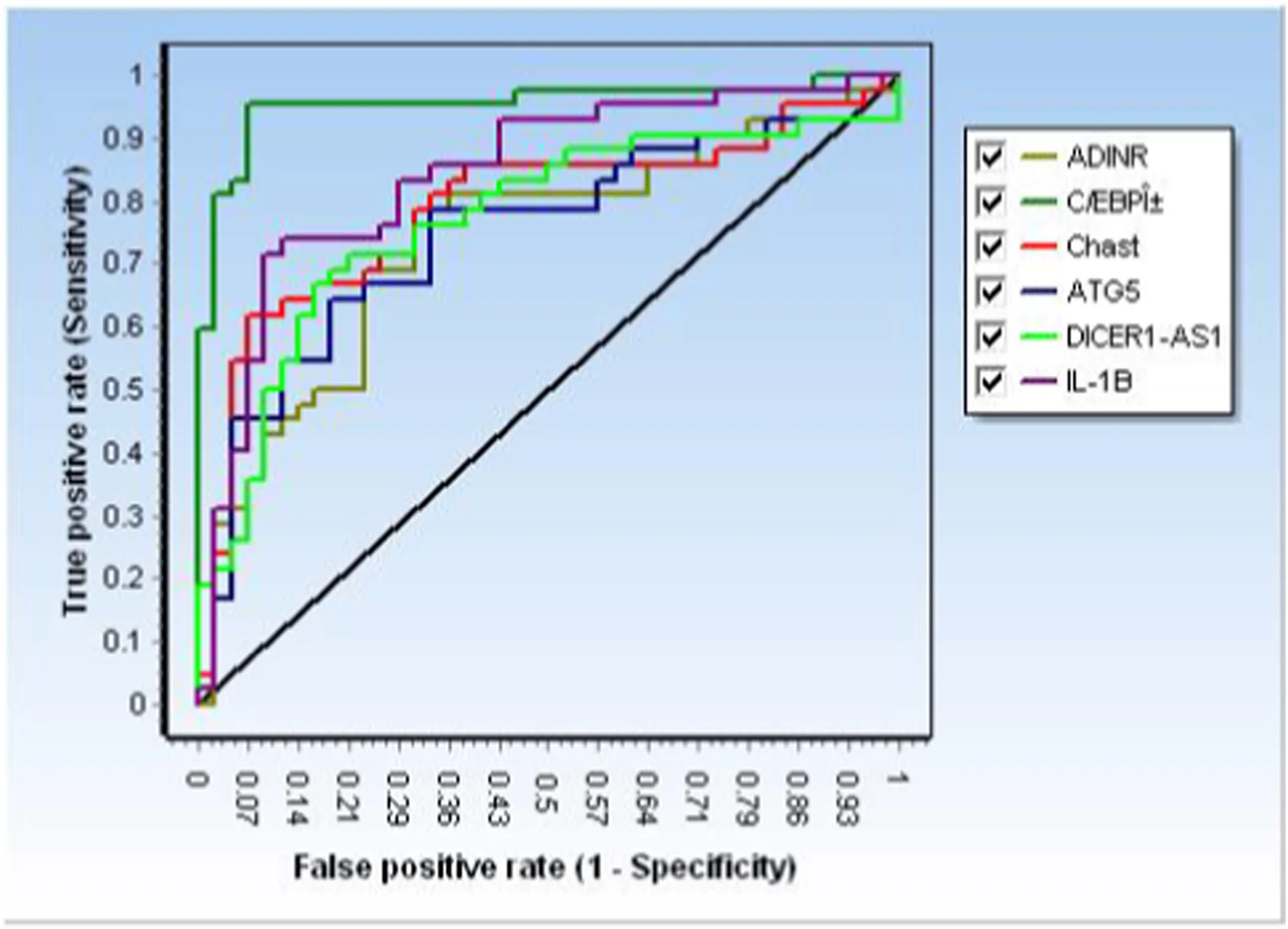
The receiver operating characteristic (ROC) curve of six upregulated genes for discrimination of Tumor tissues (TT) from Normal Tissue Adjacent to the Tumor (NTAT).

The suitability of ADINR, C/EBPα, CHAST, ATG5, IL-1B, and DICER1-AS1 for differentiation of TTs and NTATs was assessed using ROC curves (Fig. 4).

Based on the ROC curve analyses, accuracy of mentioned transcripts ranged from 94.12% for C/EBPα to 72.26% for ADINR (Table 5). The highest PPV and NPV belonged to C/EBPα (93.02 and 95.12, respectively). AUC values were 0.95, 0.84, 0.79, 0.77 and 0.74 for C/EBPα, IL-1B, CHAST, DICER1-AS1 and ADINR, respectively.

**Table 5:** Diagnostic performance of negative delta Ct cut-offs for gene expression in differentiating tumor tissues from Normal Tissue Adjacent to the Tumor: Sensitivity, Specificity, Accuracy, PPV, NPV and AUC based on ROC Curve and Youden's Index Analysis.

Gene	Cut-off ΔCt	Sensitivity (%)	Specificity (%)	Accuracy (%)	PPV (Positive Predictive Value)	NPV (Negative Predictive Value)	AUC (Area Under Curve)
C/EBPα	> −10.75	95.24 (84.2-99.1)	92.86 (81-97.5)	94.12	93.02	95.12	0.95 (0.9-1)
IL-1B	> −8.543	71.43 (56.4-82.8)	90.48 (77.9-96.2)	80.95	88.24	76	0.84 (0.75-0.93)
CHAST	> −7.849	61.9 (46.8-75)	92.86 (81-97.5)	77.38	89.66	70.91	0.79 (0.68-0.89)
DICER1-AS1	> −10.28	71.43 (56.4-82.8)	78.57 (64-88.3)	78.5	76.9	73.3	0.77 (0.66-0.87)
ADINR	> −13.01	80.95 (66.7-90.2)	64.29 (49.2-77)	72.26	80.95	64.29	0.74 (0.63-0.84)

95% confidence intervals are reported for AUC, sensitivity, and specificity.

Moreover, -delta Ct cut-offs were used for classification of TT and NTAT samples to high and low expression groups (Table 6). While most of TT samples were classified as high expressors for each gene, NTAT samples followed the opposite trend.

**Table 6:** Classification of tumor tissues from Normal Tissue Adjacent to the Tumor into high and low gene expression groups based on -delta Ct cut-offs (N (%)).

Groups	C/EBPα High	C/EBPα Low	IL-1B High	IL-1B Low	CHAST High	CHAST Low	DICER1-AS1 High	DICER1-AS1 Low	ADINR High	ADINR Low
NTAT	3 (7.14%)	39 (92.86)	4 (9.5%)	38 (90.5)	3 (7.14%)	39 (92.86)	9 (21.42%)	33 (78.58%)	15 (35.71%)	27 (64.29%)
TT	40 (95.24%)	2 (4.76)	30 (71.42%)	12 (28.58%)	26 (61.9%)	16 (38.1%)	30 (71.42%)	12 (28.58%)	34 (80.95%)	8 (19.05%)

Relative expression levels of C/EBPα and DICER1-AS1 were associated with age of patients (P values = 0.01 and 0.03, respectively). For both genes, higher levels were observed in age group of patients that were younger than 58 years (Table 7). In this study, we analyzed gene expression levels of 9 genes in 42 lung tissue samples, comprising 42 tumor and 42 adjacent normal tissues. Among the tumor samples, 37 were classified as NSCLC and 5 as carcinoid. Due to the imbalance in the sample distribution, we did not perform a statistical comparison of gene expression between these two tumor groups. However, we conducted a subtype-specific analysis of gene expression in the two major NSCLC subtypes, adenocarcinoma (n = 18) and squamous cell carcinoma (n = 14), as the sample size between these groups was relatively balanced. No significant differences in gene expression were observed between tumor subtypes.

**Table 7:** Comparison of expression levels between diverse groups. Mann-Whitney test was used for comparing gene expression levels between groups.

Parameters	Subclasses	Number of patients	Relative expression level of ADINR (mean ± SD)	P-value	Relative expression level of C/EBPα (mean ± SD)	P-value	Relative expression level of CHAST (mean ± SD)	P-value	Relative expression level of ATG5 (mean ± SD)	P-value	Relative expression level of DICER1-AS1 (mean ± SD)	P-value	Relative expression level of IL-1B (mean ± SD)	P-value
Age	<58	N = 20	−9.9 ± 2.8	0.5	−4.02 ± 3.1	**0.01**	−6.7 ± 2.7	0.31	−8.05 ± 2.1	0.34	−6.8 ± 5.7	**0.03**	−6.6 ± 2.5	0.2
	≥58	N = 22	−11 ± 3.45		−6.8 ± 3.6		−8.1 ± 4.34		−9.34 ± 3.7		−9 ± 3.9		−7.9 ± 3.8	
Subtypes	Adenocarcinoma	N = 18	−10.4 ± 2.97	0.9	−4.8 ± 3.3	0.37	−7.03 ± 3.3	0.47	−8.4 ± 2.2	0.57	−7.76 ± 4.7	0.37	−7 ± 2.7	0.69
	squamous cell carcinoma	N = 14	−10.6 ± 3.47		−6.2 ± 3.74		−7.7 ± 4		−9.1 ± 3.9		−8.3 ± 5.2		−7.6 ± 3.9	

Additionally, a Chi-square test was conducted to examine the association between NSCLC subtypes and age. A significant relationship was identified between age and subtype (P = 0.004). Specifically, 12 out of 14 SCC samples were from individuals older than 58 years, whereas only 2 were from those younger than 58. In contrast, among the 18 adenocarcinoma samples, 8 were from individuals above 58, and 10 were from those younger than 58.

## 4 Discussion

**Fig. 5:**
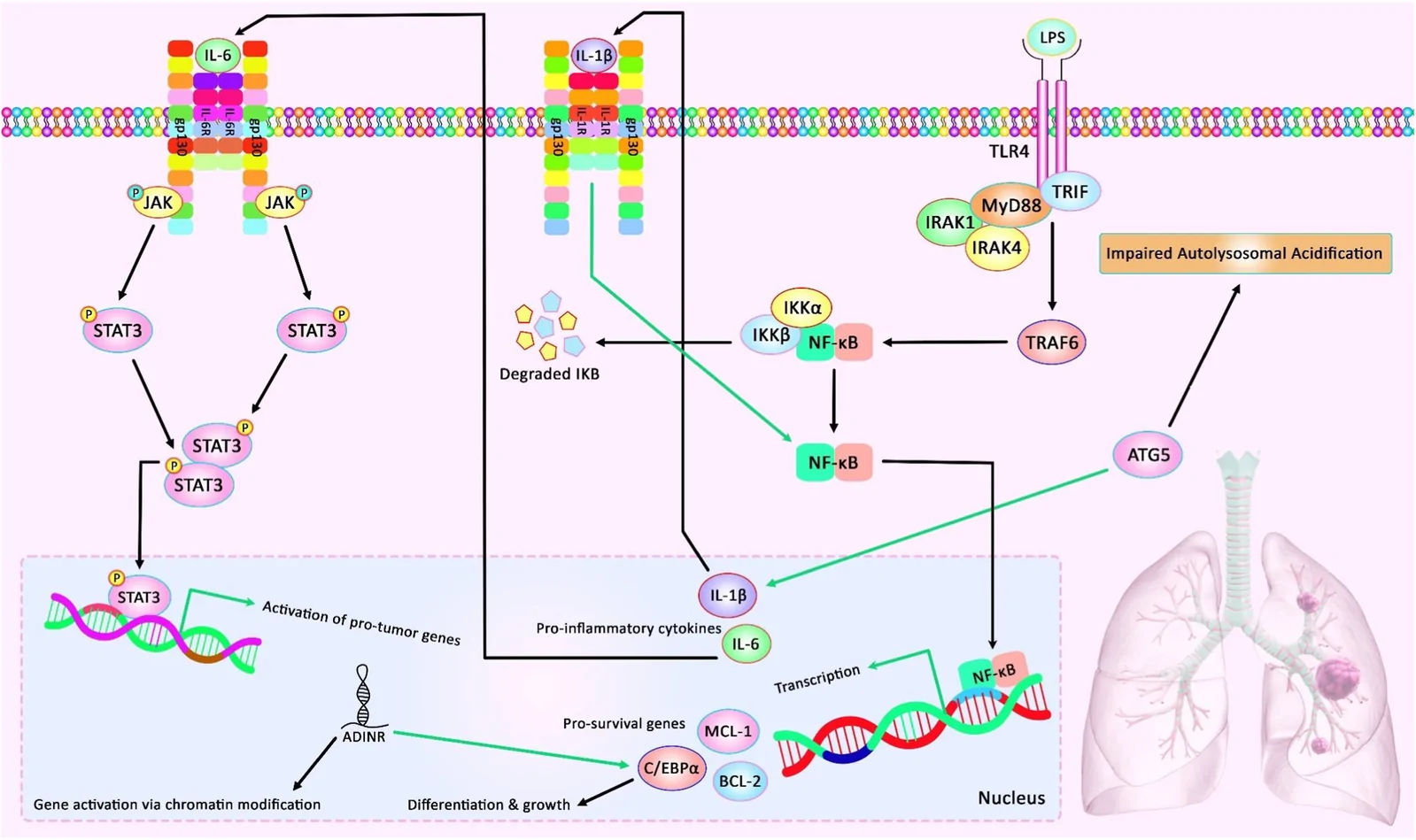
**Proposed model for LPS-TLR4/NF-κB/STAT3 signaling and regulation of transcripts in lung tumor cells.** LPS activates TLR4, inducing NF-κB nuclear translocation and transcription of pro-inflammatory, pro-survival, and autophagy genes. ADINR enhances C/EBPα transcription, promoting survival and proliferation. Secreted IL-6 activates STAT3 via IL-6R/gp130/JAKs [21], supporting survival, proliferation, angiogenesis, and immune evasion. ATG5 regulates expression of IL-1β [22]. NF-κB/STAT3 crosstalk sustains a pro-tumor microenvironment [23]. Green arrows denote upregulation. This schematic is a literature-based hypothetical model. No mechanistic interactions depicted here were experimentally tested in the present study.

NF-κB is a pleiotropic TF that affect oncogenesis. NF-κB signaling has been found to contribute to the progression and spread of lung cancer [20]. Thus, development of antagonists to this pathway has important role in avoidance of lung cancer progression [20]. Identification of the regulatory network of this TF would be a step toward design of novel therapies for lung cancer. In the current study, we examined expression of three NF-κB-associated-mRNA coding genes and five lncRNAs from this pathway in paired lung cancer samples. Notably, we found significant up-regulation of ADINR, C/EBPα, CHAST, ATG5, IL-1B, and DICER1-AS1 in TT samples compared with NTATs. While the present work performed no mechanistical studies, literature search provided some clues about the role of mentioned genes in the regulation of NF-κB–mediated pro-inflammatory and autophagic cascades. Fig. 5 illustrates a proposed model for the overexpressed transcripts associated with NF-κB–mediated pro-inflammatory and autophagic cascades in lung cancer as demonstrated in previous studies. This schematic is a literature-based hypothetical model. No mechanistic interactions depicted here were experimentally tested in the present study.

Non-coding RNAs have been recently attracted the attention of researchers for their potential in their therapeutic approaches in diverse malignant and inflammatory disorders. Thus, therapeutic approaches targeting these transcripts might affect central pathologic processes in cancers. In fact, non-coding RNA based therapeutics might pave the way for innovative therapeutic approaches in different disorders, including lung cancer, where both oncogenic and inflammatory factors are involved.

ADINR is a transcript which is transcribed from upstream of the *C/EBPα* gene and regulates its expression. In fact, ADINR has an essential role in the maintenance of active chromatin configuration of the C/EBPα. Thus, the parallel up-regulation of these transcripts is consistent with the supposed cooperation between NF-κB and C/EBPβ and the resultant induction of inflammatory mediators [24]. ATG5-mediated autophagy has been found to suppress NF-κB signaling and preclude inflammatory responses [14]. Thus, the parallel up-regulation of ATG5 and ADINR, C/EBPα, and IL-1B cannot be explained by this function of ATG5. However, it has been well-acknowledged that ATG5 has several autophagy-independent functions [25]. Among these function is the inhibitory role of ATG5 on IL-1β degradation which is exerted through disrupting autolysosomal acidification, suggestive of the non-autophagic pathway whereby ATG5 controls exosome production [26]. This function may be relevant to the observed parallel up-regulation of ATG5 and ADINR, C/EBPα, and IL-1B in lung cancer tissues.

In addition, we found significant up-regulation of DICER1-AS1 in lung cancer tissues. In line with this finding, DICER1-AS1 has been shown to promotes colorectal carcinogenesis through regulating MAPK/ERK axis [27]. As a new piece of the puzzle of NF-κB-associated interacting network, IL-1 has been demonstrated to contribute to the MAPK/ERK/NF-κB cross-talk [17]. These observations were again verified in our experiments where we showed significant correlations between expression levels of all studied mRNA coding genes and lncRNAs in both TTs and NTATs. Most notably, the highest R value belonged to ADINR/C/EBPα pair in NTATs, and IL-1B/ATG5 pairs in TTs which can be explained by the before-mentioned cross-talks between these genes.

In contrast to the six transcripts described above, we did not observe significant differential expression of ANRIL, HULC, or NKILA between two sets of samples. However, when repeating analysis for NSCLC samples only (excluding carcinoid samples), ANRIL shifted from non-significant (full cohort, q = 0.060) to significant (NSCLC-only, q = 0.0346) (Table S1). Several possible explanations may account for these negative findings. First, tissue specificity and cancer type dependence may play a role. While ANRIL has been implicated in multiple malignancies, including lung cancer, its expression patterns vary substantially across cancer types and even across subtypes within the same organ. Similarly, HULC was originally identified in hepatocellular carcinoma and has been most consistently studied in liver cancers; its role in lung cancer remains less established, and expression levels may be more variable. NKILA, an NF-κB-interacting lncRNA, has been shown to suppress NF-κB signaling by masking phosphorylation sites on IκBα. The lack of significant up-regulation in our cohort may reflect a more complex, context-dependent regulatory relationship in lung cancer compared to other cancers where NKILA dysregulation has been reported. In addition, the heterogeneity of our cohort (mixed NSCLC and carcinoid tumors) may have obscured true expression differences that might be detectable in a more histologically homogeneous population.

We also illustrated expression levels of 6 target genes in 42 paired lung tissue samples in a heatmap. ADINR, C/EBPa, CHAST, ATG5, IL-1B, and DICER1-AS1 genes demonstrated significant upregulation in TTs compared to their normal counterparts, suggesting a potential role in tumorigenesis. Four clusters were found. In brief, CHAST and ATG5 clustered in one group (Cluster 1); Cluster 2 described ADINR levels; Cluster 3 included C/EBPa and IL-1B; and Cluster 4 described DICER-AS1 levels. Most of tumor samples (TT43-TT84) were located on the right side with higher expression of studied genes in this work. Besides, DPCA revealed partial separation between NTAT and TT samples. This suggests that the differences in the expression of studied gene might be associated with the pathophysiology of lung cancer, and these genes might establish an important biomarker signature for lung cancer. These findings were also confirmed by ROC curve analyses where we showed that accuracy of mentioned transcripts in differentiation of TTs from NTATs ranged from 94.12% for C/EBPα to 72.26% for ADINR. Importantly, while ROC curve analyses showed promising AUC values, these results should be interpreted as exploratory. Diagnostic biomarker development requires multiple stages of validation, including independent replication, assessment of specificity and sensitivity against existing standards, and multivariate modeling to control for confounders — none of which were possible in the current study design.

Finally, we showed significant association between expression levels of C/EBPα and DICER1-AS1 and age of patients in a way that higher levels of these genes were observed in age group of patients that were younger than 58 years. Since age is regarded as a prognostic factor in lung cancer patients [28], the significance of this finding should be assessed in future studies. However, because the cohort was small and histological subtypes were unevenly distributed, especially with only five carcinoid cases, we did not perform multivariable regression analysis. Such adjustment would have been statistically underpowered and potentially unstable. Therefore, the observed associations between age and gene expression should be considered exploratory and may be influenced by subtype distribution.

In summary, this descriptive study reports significant up-regulation of ADINR, C/EBPα, CHAST, ATG5, IL-1B, and DICER1-AS1 in lung cancer tissues compared with NTATs. The high AUC values observed for some transcripts, particularly C/EBPα, suggest that these genes may warrant further investigation as potential tissue-based diagnostic candidates. However, the translational impact of this study is limited by lack of functional studies. In fact, due to the lack of independent validation, comparison with established markers, multivariate adjustment, and adequate subtype-specific analyses, no clinical claims can be made at this time. Future studies with larger, multicenter cohorts and prospective designs are necessary to determine whether these transcripts add value to current diagnostic approaches.

## 5 Limitations

Our study has several limitations. First and foremost, this investigation is primarily descriptive in nature. While we have identified significant differential expression of ADINR, C/EBPα, CHAST, ATG5, IL-1B, and DICER1-AS1 in lung cancer tissues compared to NTATs, the study does not provide mechanistic validation of how these transcripts contribute to lung cancer biology. Specifically, no functional experiments (such as gain- or loss-of-function studies in cell lines or animal models), pathway analyses beyond correlation, or direct molecular interactions (e.g., RNA-protein binding, chromatin immunoprecipitation, or luciferase reporter assays) were performed. Consequently, we cannot establish causality between the observed expression changes and specific oncogenic processes such as proliferation, apoptosis, invasion, or inflammation. Our findings should therefore be interpreted as hypothesis-generating rather than conclusive evidence of biological function.

Second, the relatively small sample size (n = 42) and the inclusion of both NSCLC and carcinoid tumors limit the generalizability of our findings. Due to the small number of carcinoid samples (n = 5) and the substantial biological differences between these tumor types, we did not perform statistical comparisons between the two groups. Pooling these histologically distinct entities is a clear limitation, as carcinoid tumors are neuroendocrine neoplasms with a distinct pathogenesis, clinical behavior, and molecular landscape compared to NSCLC. The inclusion of both types may have contributed to the observed expression variability and potentially confounded the results. However, we performed sensitivity analyses excluding carcinoid samples (Table S1). The direction of effect was preserved for all nine genes; however, ANRIL, previously borderline, reached significance in the NSCLC-only subgroup. Moreover, we did not compare the diagnostic performance of these transcripts with established clinical or molecular markers (e.g., histopathology, CT imaging, or currently used protein or miRNA biomarkers). Since we did not perform multivariate analyses adjusting for potential confounders such as age, smoking status, tumor stage, or comorbidities; the independent contribution of each transcript to diagnostic discrimination remains unknown. Due to incomplete stage annotation, inconsistent smoking records, and lack of follow-up data, robust clinicopathological and prognostic analyses were not possible in this cohort.

Third, we did not assess protein expression levels of the protein-coding genes (C/EBPα, ATG5, IL-1B). Therefore, whether the observed mRNA up-regulation translates into increased protein function remains unknown.

Fourth, another important methodological limitation of the present study concerns the stability of the reference genes used for qRT-PCR normalization. Although B2M and TBP were used as housekeeping genes, geNorm analysis yielded M-values higher than commonly recommended stability thresholds, indicating appreciable variability in reference-gene expression across the studied tissue groups (Supplementary material). Additional housekeeping gene candidates could not be evaluated in the retained samples due to insufficient residual RNA/cDNA volume and concentration after the primary gene expression analyses were completed. The remaining material was below the threshold required for reliable amplification of these additional targets, and therefore these candidates were excluded from the normalization assessment. An independent assessment based on the standard deviation and coefficient of variation of raw Ct values likewise indicated that the available reference genes were not ideally stable across tumor and adjacent-normal tissues. Therefore, the absolute magnitude of the reported relative expression changes should be interpreted with some caution, as variation in reference-gene expression may influence the estimated fold changes. Importantly, however, normalization using B2M alone, TBP alone, or their geometric mean resulted in consistent directions of differential expression across the examined target genes. Thus, although reference-gene instability may affect the magnitude of the quantitative estimates, the overall direction of the observed transcriptional alterations was relatively robust to the normalization strategy. Future studies should validate a larger panel of candidate reference genes and identify the most stable normalization factor specifically for tumor and matched adjacent-normal tissues.

Finally, while we propose these transcripts as potential tissue biomarkers based on ROC analyses, their specificity and sensitivity need to be evaluated in larger, independent patient cohorts and compared against existing clinical biomarkers. Because ROC cut-offs were derived and tested in the same cohort, the reported thresholds may overestimate performance and should be validated in an independent external cohort. Prospective studies are also required to determine whether expression levels correlate with patient prognosis, treatment response, or disease progression.

Despite these limitations, the current study provides a foundational descriptive resource for NF-κB-related pro-inflammatory and autophagic transcripts in lung cancer, which can guide future mechanistic and translational investigations.

## Ethics declaration

Written informed consent to take part in the study and to publish the article has been obtained from all participants or their legal representatives. The privacy rights of participants have been observed.

This study included organ or tissue donors. This study includes human biological material and consent was obtained by donors, or their next of kin or legal representatives, for use in this study and for publication of the article. The samples used in this research were not sourced from executed prisoners or prisoners of conscience.

This study was performed in compliance with relevant laws, regulatory frameworks and guidelines where the research took place. This study was approved by the Shahid Beheshti University of Medical Sciences. (Approval No. IR.SBMU.RETECH.REC.1404.833)

## Ethics approval

All methods were in agreement with the ethical guidelines of the national research committee and with the 1964 Helsinki declaration and its subsequent modifications. Informed consent forms were acquired from all participants. The study protocol was verified by the ethical committee of Shahid Beheshti University of Medical Sciences.

## Consent of publication

Not applicable.

## Use of generative AI and AI-assisted technologies

During the preparation of this work, the authors used DeepSeek in order to enhance the readability of the manuscript. After using this tool/service, the authors reviewed and edited the content as needed and take full responsibility for the content of the published article.

## Role of the funding

The current study was supported by a grant from Shahid Beheshti University of Medical Sciences.

## Declaration of competing interest

The authors declare that they have no known competing financial interests or personal relationships that could have appeared to influence the work reported in this paper.

## Data Availability

All data generated or analyzed during this study are included in this published article and its supplementary information files.
