# Supplementary material for: Pro-inflammatory and autophagic pathways in lung cancer: dysregulation of key genes ADINR, C/EBPα, CHAST, ATG5, IL-1B, and DICER1-AS1
Source: Biochem Biophys Rep. 2026 Sep 8;48:102774. doi: 10.1016/j.bbrep.2026.102774 (PMC13573000; doi:10.1016/j.bbrep.2026.102774)
Supplement: Supplementary file 1 [file mmc1.docx]

Table S1. Sensitivity analysis restricted to patients with non-small cell lung cancer (NSCLC).

| **Gene** | **Expression ratio** | **95% CI** | **P-value** | **q-value** |
| --- | --- | --- | --- | --- |
| ANRIL | 2.42 | 0.88-6.65 | 0.08 | 0.0346 |
| ADINR | 6.7 | 2.87-15.6 | <0.0001 | <0.0001 |
| C/EBPα | 490.4 | 204.5-1176 | <0.0001 | <0.0001 |
| CHAST | 11.7 | 4.2-32.7 | <0.0001 | <0.0001 |
| ATG5 | 6.05 | 2.42-15.1 | 0.0002 | 0.0001 |
| DICER1-AS1 | 13.3 | 4.23-41.8 | <0.0001 | <0.0001 |
| HULC | 1.6 | 0.56-4.5 | 0.36 | 0.1364 |
| IL-1B | 34.5 | 10.9-108.9 | <0.0001 | <0.0001 |
| NKILA | 0.82 | 0.28-2.37 | 0.58 | 0.1953 |
